# Comparative efficacy different resistance training protocols on bone mineral density in postmenopausal women: A systematic review and network meta-analysis

**DOI:** 10.3389/fphys.2023.1105303

**Published:** 2023-02-07

**Authors:** Zhenyu Wang, Xingchun Zan, Yongjie Li, Yue Lu, Yuan Xia, Xinyong Pan

**Affiliations:** ^1^ School of Health Sciences, Wuhan Sports University, Wuhan, China; ^2^ Department of Hyperbaric Oxygen, The Second People’s Hospital of Hefei, Hefei Hospital Affiliated to Anhui Medical University, Hefei, China; ^3^ Department of Rehabilitation Medicine, Beijing Jishuitan HospitalGuizhouHospital, Guiyang, China; ^4^ Department of Rehabilitation Medicine, Hubei Provincicial Hospital of Integrated Chinese and Western Medicine, Wuhan, China

**Keywords:** resistance training, bone mineral density, postmenopausal, meta analysis, protocol

## Abstract

**Objective:** To systematically review the effects of different resistance training (RT) protocols on bone mineral density (BMD) in postmenopausal women.

**Methods:** Randomized controlled trials (RCTs) on the resistance training in improving bone mineral density for postmenopausal women were searched in databases including ProQuest, PubMed, Cochrane Library, Embase, and Web of Science. The retrieval time range was from the establishment of the database to May 2022. The included literature was independently screened and relevant data was extracted by two reviewers. The systematic review followed the Joanna Briggs Institute (JBI) methodology for reviews of quantitative evidence. Quality of risk was assessed using the Physical Therapy Evidence Database (PEDro) scale, risk of bias was assessedusing the Cochrane RoB2 tool and a network Meta-analysis was performed on the data using Stata 16.0.

**Results:** A total of 19 studies, which included 919 subjects, were eventually acquired. The results of the network Meta-analysis showed that moderate intensity resistance training was superior in improving lumbar spine bone mineral density (LS BMD) and femoral neck bone mineral density (FN BMD) compared to the control group (as per usual daily life), with a statistically significant difference (*p <* 0.05). There was, however, no statistically significant difference between the groups in terms of increasing total hip bone mineral density (TH BMD) and trochanter bone mineral density (Troch BMD), although moderate intensity training tends to increase bone mineral density (*p >* 0.05). In addition, when training frequency is taken into consideration, 3 days/week of moderate intensity training (3MI) was superior to 2 days/week (2MI) in improving lumbar spine bone mineral density , and moderate intensity training was superior to low and high intensity resistance trainings at training frequency of 3 day/week, with statistically significant differences (*p <* 0.05). The cumulative probability ranking results indicated that 3MI was the optimal option in improving lumbar spine, femoral neck, total hip and Troch bone mineral density. Subgroup analyses combining interventions time showed that for lumbar spine and femoral neck bone mineral density, 3MI protocol with intervention duration within 1 year (≤48 weeks) had a significant advantage over other interventions, while this advantage was no longer significant with the intervention duration of more than 1 year (>48 weeks).

**Conclusion:** Current evidence shows that moderate intensity resistance training for 3 days/week can be preferred clinically to improve bone mineral density in postmenopausal women, and it is recommended that the duration of the same training should not exceed 1 year. Nevertheless, more high-quality studies are needed to verify the above conclusion.

## 1 Introduction

Bone loss is significantly accelerated with the loss of estrogen after menopause in elderly women. Studies have shown that during 1–10 years after menopause, the annual loss rate of human bone mass is 1.5%–2.5% (Cheng et al., 2020). BMD and bone mass reduction above a certain range is prone to osteoporosis, which increases the risk of fracture by 2.6 times (Sathyapalan et al., 2017). Approximately 200 million women worldwide suffer from osteoporosis after menopause, because the destruction of trabecular bone structure results in increased bone fragility and decreased bone mechanical strength, which consequently adds the risk of fracture (Levin et al., 2018). Osteoporosis and fall risk are determinants of fragility fractures. According to the Iolascon study, women with higher fall risk exhibit more osteoporotic fractures and poorer physical performance, leading to more medication intake. Besides, patients with osteoporosis and fractures are more prone to disability and highly dependent on others in daily activities, which significantly undermines their life qualities (Global Burden of Disease Study 2013 Collaborators, 2015). Accordingly, it is essential to find ways that effectively prevent and treat osteoporosis (Reginster and Burlet, 2006; Jackson and Mysiw, 2014). Despite the effective role of drugs that improve bone mineral density (BMD) in treating osteoporosis (Crandall et al., 2014), the non-adherence and lack of persistence of anti-osteoporosis therapy lead to poor outcomes in practice, such as less reduction in bone turnover rate, smaller increase in BMD, and a significant rise in fracture risk (Migliaccio et al., 2013; Ringe and Farahmand, 2014; Lagari et al., 2015).

It is proved that exercise training, such as weight bearing, progressive resistance training, strength training, etc., can significantly reduce fall rate to improve function recovery and decrease fracture risk (Benedetti et al., 2018). In addition, the theoretical basis for osteoporosis prevention is to furthest increase bone mass during the peak period of bone mass balance and maintain it for a longer time. Meanwhile, physical exercise is required during the period of rapid bone mass loss to slow down its loss rate. Accordingly, exercise training for the elderly should be strengthened to prevent bone loss and increase BMD, so as to effectively prevent osteoporosis and reduce fall risk. The Clinical Practice Guidelines (CGP) advocates exercise of moderate-to-high intensity to prevent bone loss (Füzéki and Banzer, 2018), which, however, differs in recommended exercise intensity, and fails to make specific indications on the type, frequency, intensity, and duration of exercise. Therefore, relevant investigation should be conducted to improve the quality of evidence regarding the application of this intervention in the management of patients with osteoporosis. Previous studies have demonstrated the obvious advantages of resistance training (RT) over other exercise methods in increasing BMD (Palombaro et al., 2013), however, no consensus has been reached on the optimal training intensity and frequency. Therefore, this study designed various combinations of training intensity and frequency using a network Meta-analysis to find the optimal scheme, so as to compare the effects of different schemes on BMD in postmenopausal women, and provide a scientific basis for exercise prescriptions that can improve BMD in the elderly population.

## 2 Materials and methods

The systematic review of this paper was conducted according to the Preferred Reporting Items for Systematic Review and Meta-Analyses (PRISMA); this study has been registered with the International Prospective Registerof Systematic Reviews (PROSPERO) under the registration number: CRD42020212253.

### 2.1 Inclusion and exclusion criteria

Using the PICO (Population/patients, Intervention/exposure, Control/comparison, and Outcome) strategy, the studies that meet the following criteria were included in the study. Based on the previous studies (Kistler-Fischbacher et al., 2021), the training intensity was divided into high intensity (≥80% 1RM), moderate intensity (65%–80% 1RM) and low intensity (≤65% 1RM), and frequency was divided into high frequency (3 days/week) and low frequency (2 days/week). The PICO strategy is presented in Table 1.

**TABLE 1:** Formulated question of the study based on PICO(S).

Components of PICO(S)	Defined as
Population/patients	Postmenopausal women (>50 years)
Intervention/exposure	Received one of the following resistance trainings: High intensity and high frequency resistance training (3HI), moderate intensity and high frequency resistance training (3MI), lowintensity and high frequency resistance training (3LI), high intensity and low frequency resistance training (2HI), moderate intensity and low frequency resistance training (2MI), and low intensity and low frequency resistance training (2LI)
Control/comparison	Only lived daily life and did not receive any additional training interventions
Outcome	LS, FN, TH, and Troch BMD were taken as the outcome measures and dual-energy X-ray absorptiometry (DXA) acted as the test method
Study design	Randomized controlled trials

The exclusion criteria adopted in the present study are as follows:

(1) Concurrent drug therapy during resistance training;
(2) Mixed combination of exercise interventions apart from resistance training;
(3) Had undergone a high intensity physical training in the 6 months prior to the trial;
(4) Exercise intervention intensity not marked in detail or intensity expressed in a non-RM way;
(5) Liver, kidney and endocrine system diseases that affecting bone metabolism;
(6) Abstracts, reviews, conference reports, etc.;
(7) Multiple publications of the literature;
(8) Non-extractable data;
(9) Full text not available;
(10) Lack of outcome measures

### 2.2 Data sources and retrieval strategies

In this study, the search strategy was developed and implemented according to PRISMA. A systematic search was conducted in databases including ProQuest, PubMed, Cochrane Library, Embase, and Web of Science with a retrieval time range from the establishment of the database to May 2022.

The search terms included bone mineral density (BMD), resistance training, strength training, and postmenopausal. The specific search strategy was as follows (PubMed was used as an example): (Bone Mineral Density OR Density, Bone OR bone density OR BMD) AND (exercise OR resistance training OR resistance OR weight lift OR strength training OR strength exercise OR weight training) AND (post-menopausal OR post menopause OR menopausal).

### 2.3 Data collection and extraction

After duplicate data was removed using EndnoteX9, two reviewers independently analyzed the titles and abstracts of all literature retrieved from the database. Then, they browsed through the entire articles, further screened out those that met the inclusion criteria, downloaded the full text, and divided them into three groups-included, possibly included, and excluded. For any disagreement, articles were reevaluated. If disagreements persisted, group discussions were held to resolve it.

The following references were extracted from each study by two reviewers using pre-assigned tables, including the first author, year of publication, country, intervention time, sample size (enrollment/disenrollment), mean age of participants, exercise intervention plan (group count setting, frequency, intensity, periodicity and Resistance training type) and outcome measures, the main result and complication.

### 2.4 Risk of bias

Methodological quality and risk of bias of the included studies were assessed using the Joanna Briggs Institute (JBI) Checklists Critical Appraisal Tool for Randomized Controlled Trials (RCTs) (Sukan et al., 2022), PEDro scale (Le et al., 2014) and Cochrane RoB2 (Minozzi et al., 2020). The PEDro scale has been widely used to classify the quality of evidence in randomized controlled trials, aiming to enhance application of the best evidence in clinic treatment to make physical therapy more effective. The PEDro scale is a RCT rating scale based on the Delphi list (Maher et al., 2003). It includes 11 items, each item is worth one point, and the total score is 10 points (the final score excludes any “inclusion criteria” item), from 0 (high risk of bias) to 10 (low risk of bias), where a value ≥ 6 represents the critical value for low risk of bias studies. The risk of bias in the studies was classified into three types such as low risk, uncertain risk, and high risk according to the JBI assessment results. Based on the number of “yes” responses, <40% was considered “high risk,” 40%–80% “moderate risk,” and >80% “low risk. “The risk of bias was assessed using Cochrane RoB 2 (Sterne et al., 2019). Five domains were included in this tool to assessthe risk of bias, including bias because of 1) randomizationprocess, 2) deviations from intended interventions, 3) missing outcome data, 4) measurement of the outcome, and 5) selection of the reported results. Each domain could bescored as low, moderate, or high risk of bias. Finally, an overall risk of bias score was provided.

### 2.5 Statistical analysis

Data preprocessing and analysis were performed by two reviewers. The raw data was preprocessed using Microsoft Office Excel and results were indicated as the difference value between the endpoint and base values. All results were converted into a mean value and its standard deviation (ΔMean ± SD) in uniform unit of g/cm^2^.

Network Meta-analysis and graphical plotting were performed for relevant data using Stata 16.0 software. The outcome measures were continuous variables and rated by the same scale, so the weighted mean difference (WMD) and 95% confidence interval (CI) were taken as effect sizes. The network evidence diagram of direct comparison between intervention intensity-frequency combinations was plotted; then the consistency of each outcome indicator’s closed loop was evaluated using the loop inconsistency test, where it indicated a good consistency between the direct and indirect evidence if 95% CI of the loop inconsistency factor (IF) contained 0 (Jackson et al., 2016). The results of network Meta-analysis were presented through pairwise comparison of forest plots. Cumulative ranking probability plots drawn based on the surface under the cumulative ranking curve (SUCRA) were used to determine the optimal training intensity and frequency. Comparison-correction funnel plots were used to test for publication bias and small sample effect.

Apart from the above, stability of the study results was verified using subgroup and sensitivity analyses. Subgroup analyses was performed according to the duration of intervention, while sensitivity analyses was performed by excluding studies with a sample size of less than 10.

## 3 Results

### 3.1 Search results

**FIGURE 1:**
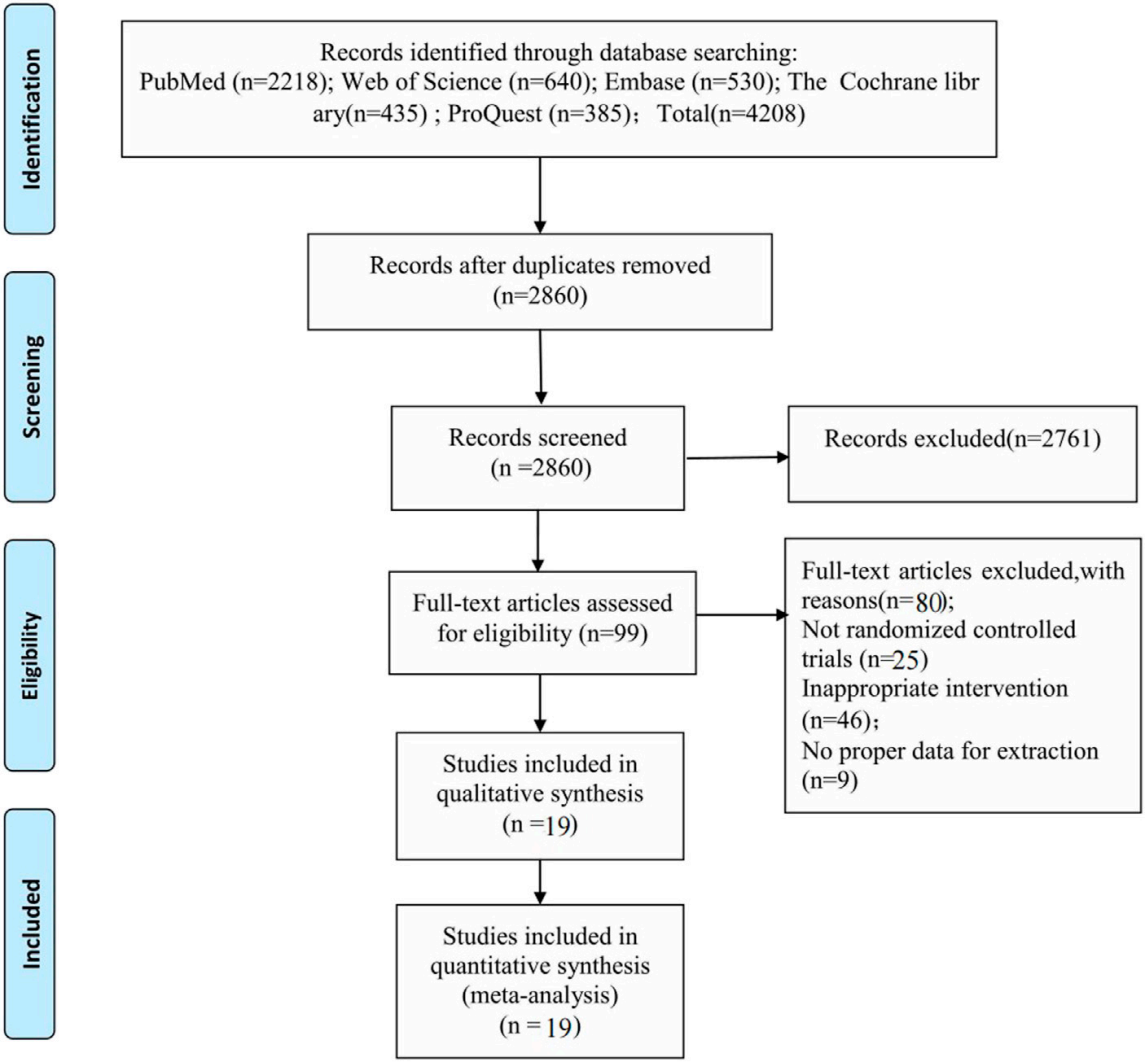
Flowchart of the study selection process.

A total of 4,208 studies were retrieved, and based on multiple screenings, 19 studies with a total of 919 patients were finally included. The flow chart of selection process in this study is shown in Figure 1.

### 3.2 Characteristics of the included studies

A total of 19 studies (Pruitt et al., 1992; Nelson et al., 1994; Nichols KPN et al., 1995; Pruitt et al., 1995; Hartard et al., 1996; Kerr et al., 1996; Bemben et al., 2000; Maddalozzo and Snow, 2000; Rhodes et al., 2000; Chilibeck et al., 2002; Milliken et al., 2003; Liu-Ambrose et al., 2004; Siegrist CL, 2006; Maddalozzo et al., 2007; Bocalini et al., 2009; Chuin et al., 2009; Bemben et al., 2010; Bocalini et al., 2010; Marques et al., 2012) with 919 subjects were included in this study. Countries where the studies were published involved the United States (n = 9), Brazil (n = 2), Australia (n = 2), Canada (n = 4), Germany (n = 1), and Portugal (n = 1). In the intervention group, eight studies involved 3HI, five involved 3MI, six involved 3LI, two involved 2HI, three involved 2MI, and 0 involved 2LI. BMD of four sites were included in the outcome measures, respectively LS (n = 16), FN (n = 19), TH (n = 8) and Troch (n = 8). The detailed basic characteristics of the included studies are as shown in Table 2.

**TABLE 2:** The characteristics of included studies.

Included study	Country	Sample capacity	Age	Intervention measures	Intervention number	Intervention intensity	Intervention frequency	Period	Outcome measures
		Entry/fall off			set × times		day × min/w		
Pruitt et al. (1992)	United States	17	LI:53.6 ± 1.0	LI/CT	1 × 10	50%–60%1RM	3 × 60/w	36 weeks	LS、FN
		10	CT:55.6 ± 0.9						
Nelson et al. (1994)	United States	21/1	HI: 61.1 ± 3.7	HI/CT	3 × 8	80%1RM	2 × 45/w	52 weeks	LS、FN
		19/0	CT: 57.3 ± 6.3						
Nichols KPN et al. (1995)	United States	17/3	HI: 67.8 ± 1.6	HI/CT	1 × 8	80%1RM	3 days/w	48 weeks	LS、FN、Troch
		17/3	CT: 65.2 ± 1.2						
Pruitt et al. (1995)	United States	15/7	HI: 67.0 ± 0.5	HI/LI/CT	2 × 7	80%1RM	3 × 50–55/w	48 weeks	LS、FN、TH
		13/6	LI: 67.6 ± 1.4		1 × 14	40%1RM	3 × 50–55/w		
		12/1	CT: 69.6 ± 4.2						
Hartard et al. (1996)	Australia	18/2	MI: 63.6 ± 6.2	MI/CT	(Sathyapalan et al., 2017; Cheng et al., 2020) × 8–12	70%1RM	2 days/w	24 weeks	LS、FN
		16/1	CT: 67.4 ± 9.7						
Kerr et al. (1996)	Australia	28/3	MI: 58.4 ± 3.7	MI/LI	3 × 8	75%–80%1RM	3 × 20–30/w	48 weeks	FN、Troch
		28/7	LI: 55.7 ± 4.7		3 × 20	60%–65%1RM	3 × 45–60/w		
Bemben et al. (2000)	United States	13/3	HI: 50.5 ± 2.0	HI/LI/CT	3 × 8	80%1RM	3 × 60/w	24 weeks	LS、FN、TH
		11/4	LI: 51.9 ± 2.3		3 × 16	40%1RM	3 × 60/w		
		11/3	CT:52.3 ± 1.4						
Maddalozzo and Snow (2000)	United States	13/9	HI:54.9 ± 3.3	HI/LI	3 × 10−13	70%–90%1RM	3 × 60/w	24 weeks	LS、FN、TH
		12/9	LI:54.4 ± 3.4		3×2–10	40%–60%1RM	3 × 60/w		
Rhodes et al. (2000)	Canada	22/2	MI: 68.8 ± 3.2	MI/CT	3 × 8	75%1RM	3 × 60/w	48 weeks	LS、FN、Troch
		22/4	CT: 68.2 ± 3.5						
Chilibeck et al. (2002)	Canada	45/9	MI:54.1 ± 3.7	MI/CT	2 × 20	70%1RM	2 × 25/w	48 weeks	LS、FN、TH、Troch
		36/0	CT:50.8 ± 4.7						
Milliken et al. (2003)	United States	91/20	MI: 55.8 ± 4.7	MI/CT	2×6–8	70%1RM	3 days/w	48 weeks	LS、FN、Troch
		70/11	CT: 57.1 ± 5.0						
Liu-Ambrose et al. (2004)	Canada	34/2	HI: 79.6 ± 2.1	HI/CT	2×6–8	75%–85%1RM	2 × 50/w	25 weeks	FN、TH
		34/2	CT: 79.5 ± 3.2						
Siegrist CL (2006)	Germany	26/6	MI: 60.6 ± 4.8	MI/CT	8 × 12	60%–80%1RM	2 × 45/w	48 weeks	LS、FN
		20/1	CT: 61.4 ± 4.7						
Maddalozzo et al. (2007)	United States	35/6	MI: 52.3 ± 3.3	MI/CT	3×8–12	60%–75%1RM	3 × 60/w	48 weeks	LS、FN、TH、Troch
		34/5	CT: 52.5 ± 3						
Bocalini et al. (2009)	Brazil	23/8	HI: 69.0 ± 34.8	HI/CT	3 × 10	85%1RM	3 × 60/w	24 weeks	LS、FN
		12/2	CT:67.0 ± 25.2						
Chuin et al. (2009)	Canada	11	HI:65.4 ± 3.5	HI/CT	3 × 8	80%1RM	3 × 60/w	24 weeks	LS、FN
		7	CT:67.4 ± 3.8						
Bemben et al. (2010)	United States	22	HI: 64.0 ± 4.22	HI/CT	3 × 10	80%1RM	3 days/w	32 weeks	LS、FN、TH、Troch
		12	CT:63.1 ± 4.85						
Bocalini et al. (2010)	Brazil	13	MI:66.0 ± 9.0	MI/CT	3 × 10−12	60%–70%1RM	3 × 60/w	24 weeks	LS、FN
		12	CT:64.0 ± 8.0						
Marques et al. (2012)	Portuguesa	23/8	HI: 67.3 ± 5.2	HI/CT	2×6–8	75%–80%1RM	3 × 60/w	32 weeks	FN、TH、Troch
		24/4	CT: 67.9 ± 5.9						

Included study	Intervention measures	The main result	Resistance training type	Complication
Pruitt et al. (1992)	LI/CT	LI:LS↑ FN↓	Universal gym equipment, free weights, and ankle weights were used to perform the weight-lifting exercises	None
		CT:LS↓ FN↓		
Nelson et al. (1994)	HI/CT	HI:LS↑FN↑	The following exercises were used for training: Hip extension, kneeex tension, lateral pull-down, back extension, and abdominal flexion using pneumatic resistance machines (KeiserSports Health Equipment, Fresno, Calif)	None
		CT:LS↓FN↓		
Nichols KPN et al. (1995)	HI/CT	HI:LS↓FN↑Troch↑	Exercises: Leg flexion and extension, back extension, trunk flexion, bench press, lat pulldown, shoulder press, and seated row	None
		CT:LS↑FN↑Troch↑		
Pruitt et al. (1995)	HI/LI/CT	HI:LS↑FN↓TH↑	Nautilus and universal gym equipment were used by both groups to perform the following exercises: Bench press, lateral pulldown, military press, biceps curl, knee extension, knee flexion, hipabduction, hip adduction, leg press, and back extension	None
		LI:LS↑FN↑TH↑		
		CT: LS (−) FN↑TH↑		
Hartard et al. (1996)	MI/CT	MI:LS↑FN↓	Major groups of muscles were trained by means of the following clearly defined movements: 1) Shoulder joint, anteflexion (pectoralis major muscle), antagonistic movement of the muscles of the scapular region, abduction and adduction of the humerus; 2) hip joint, flexion andextension (psoas muscles, long head of quadriceps femoris, gluteus maximus) and abduction and adduction of the femur (for these exercises attraction machine was used, in addition, a leg press was used while the individual was sitting to exercise the quadriceps and gluteus maximus muscles by hip and knee extension); 3) trunk, abdominal muscles, erector trunci, and the other muscles of the thoracic and lumbar spine were exercised on two individually adjustable, custom-madeexercise benches	Osteoporosis
		CT:LS↓FN↓		
Kerr et al. (1996)	MI/LI	MI: FN(−) Troch↑	Both free weights and resistance machines were used. The exercises used on the exercising limb were 1) upper limb-biceps curl, wrist curl, reverse wrist curl, triceps extension (with pulley), and forearm pronation and supination (with dumbbell), and 2) lower limb-leg press, hip abduction and adduction, hamstring curl, hip flexion, and hip extension	None
		LI: FN↑Troch↑		
Bemben et al. (2000)	HI/LI/CT	HI:LS↓FN↓TH↑	The subjects performed three sets of the following exercises using Cybex (Ronkonkoma, NY) isotonic resistance training equipment: Quadriceps extension, hamstring flexion, leg press, shoulder press, biceps curl, triceps extension, seated row, and latissimus pull	None
		LI:LS↓FN↓TH↓		
		CT:LS↓FN↓TH↓		
Maddalozzo and Snow (2000)	HI/LI	HI:LS↑FN↑TH↑	The functional standing free-weight program consisted of 12 exercises: free weight back squat, deadlift, bicepscurls, sit-ups and triceps extensions and resistance machine exercises (Hammer Strength), chest press, incline chest press, shoulder press, high lat pull down, leg curl, gripper (wrist strength), and calfraise	None
		LI:LS↓ FN↑TH↑		
Rhodes et al. (2000)	MI/CT	MI: LS↑FN↑Troch↑	The circuitincluded large muscle exercises for example, chest press, leg press, biceps curl, tricepsextension, quadriceps curl, hamstrings curl. All exercises were performed on a universal gym	None
		CT: LS (−) FN↓Troch↓		
Chilibeck et al. (2002)	MI/CT	MI:LS↓FN↓TH↓Troch↑	Bench press, latissimus dorsipull-down, shoulder press, biceps curl, back extension, and hip extension, flexion, adduction, and abduction using Lever equipment (Pulse Fitness Systems, Winnipeg, Man.), as well as knee flexion, knee extension, and leg press using hammer strength equipment	Osteopenia
		CT:LS↓FN↓TH↓Troch↓		
Milliken et al. (2003)	MI/CT	MI:LS↑FN↑	The leg press, squat, seated one-arm dumbbell (alternating arms) presses, back extension, rotary torso, seated rows, and lateral pull-downs were performed to focus on the large muscles of the legs, shoulders, arms, and back	Osteopenia
		CT:LS↓FN↓		
Liu-Ambrose et al. (2004)	HI/CT	HI:FN↑TH↑	Both a Keiser^®^ Pressurized Air system (Keiser Corporation, Fresno, CA, United States) and free weights provided the training stimulus	Osteoporosis or osteopenia
		CT:FN↑TH↑		
Siegrist CL (2006)	MI/CT	MI:LS↓FN↓	Exercises: lat pulldown, rowing, chest press, butterfly, back extension, hip abduction and adduction, leg press	None
		CT:LS↓FN↓		
Maddalozzo et al. (2007)	MI/CT	MI:LS↑FN↓TH↓Troch↑	Free weight back squat and free weight	None
		CT:LS↓FN↓TH↓Troch↓	Deadlift-exercises. Deadlift exercise: The deadlift is an exercise where one lifts a loaded barbell off the ground from a stabilized bent-over position with emphasis on the muscles of the lower and upper back and legs	
Bocalini et al. (2009)	MI/CT	MI: LS↑FN↑Troch↑	Each session included the following exercises: Leg press, chest press, leg curl, latissimus pull down, elbow flexion, elbow extension, leg extension, upper back row, military press, hip abductor, hip adductor, and abdominal curls	None
		CT: LS (−) FN↓Troch↓		
Chuin et al. (2009)	HI/CT	HI:LS↓FN↓	Resistance training comprising exercises targeting large and small muscle groups including abdominals: leg press, bench press, leg extension, shoulder press, sit up, seate drow, triceps extension, and biceps curl	None
		CT:LS↓FN↑		
Bemben et al. (2010)	HI/CT	HI:LS↓FN↓TH↓Troch↓	Supine two leg press, hip flexion, hip extension, hip abduction, hip adduction, seated military press, lat pull down, and seated row	None
		CT:LS↓FN↓TH↓Troch↓		
Bocalini et al. (2010)	MI/CT	MI:LS↑FN↑	Each session included the following isotonic exercises: Legpress, leg extension, leg curl, chest press, elbow flexion, elbow extension, upper back row, and abdominal flexion	None
		CT:LS↓FN↓		
Marques et al. (2012)	HI/CT	HI:FN↓TH↑Troch↑	The RE protocol aimed to develop musclemass and strength in the following muscle groups: 1) quadriceps (leg press and leg extension), 2) hamstrings (seated leg curl),3) gluteal (hip abduction), 4) trunk and arms (double chest press, lateral raise and overhead press), and 5) abdominal wall (abdominalmachine). Subjects exercised on variable resistance machines (Nautilus Sports/Medical Industries, Independence, VA)	None
		CT:FN↓TH↓Troch↓		

Table Note: CT, control group; LI, low intensity resistance exercise; MI, moderate intensity resistance exercise; HI, high intensity resistance exercise; LS BMD, lumbar bone mineral density; FN BMD, femoral neck bone mineral density; TH BMD, total hip bone mineral density; Troch BMD, trochanter bone mineral density.

### 3.3 Quality evaluation

Table 3 provides the quality evaluation results of the included studies. In PEDro rating scale, an article was considered “good” if it was rated six scores or more. In this systematic review, the median PEDro score of the included studies was 5, within the range of (4–7), and 10 of the 19 studies scored at the predetermined level (≥6). All studies stated the inclusion criteria, All the studies were randomly assigned subjects; no studies revealed detailed allocation concealment; all studies were comparable at baseline, no studies blinded the researchers and subjects, four blinded the outcome raters, nine had a clinical dropout rate >15%, and three contained no intentional analysis. Added to that, between-group statistics, point measures, and difference value statistics were conducted on all the included studies.

**TABLE 3:** The Physical therapy evidence database (PEDro) scale.

Study	Eligib-ility criteria	Rando-mized allocation	Blinded allocat-ion	Group homo-geneity	Blinded subjects	Blinded thera-pists	Blinded assessor	Drop out <15%	Intention to-treat analysis	Between group comparison	Pointestimates and variability	PEDro score
Pruitt et al. (1992)	●	●	○	●	○	○	○	●	●	●	●	6
Nelson et al. (1994)	●	●	○	●	○	○	●	●	●	●	●	7
Nichols KPN et al. (1995)	●	●	○	●	○	○	○	○	●	●	●	5
Pruitt et al. (1995)	●	●	○	●	○	○	○	○	●	●	●	5
Hartard et al. (1996)	●	●	○	●	○	○	○	●	○	●	●	5
Kerr et al. (1996)	●	●	○	●	○	○	○	●	●	●	●	6
Bemben et al. (2000)	●	●	○	●	○	○	○	○	●	●	●	5
Maddalozzo and Snow (2000)	●	●	○	●	○	○	○	○	●	●	●	5
Rhodes et al. (2000)	●	●	○	●	○	○	○	●	○	●	●	5
Chilibeck et al. (2002)	●	●	○	●	○	○	○	●	●	●	●	6
Milliken et al. (2003)	●	●	○	●	○	○	○	○	●	●	●	5
Liu-Ambrose et al. (2004)	●	●	○	●	○	○	●	●	●	●	●	7
Siegrist CL (2006)	●	●	○	●	○	○	○	○	●	●	●	5
Maddalozzo et al. (2007)	●	●	○	●	○	○	○	○	●	●	●	5
Bocalini et al. (2009)	●	●	○	●	○	○	●	○	●	●	●	6
Chuin et al. (2009)	●	●	○	●	○	○	○	●	●	●	●	6
Bemben et al. (2010)	●	●	○	●	○	○	○	●	●	●	●	6
Bocalini et al. (2010)	●	●	○	●	○	○	●	●	●	●	●	7
Marques et al. (2012)	●	●	○	●	○	○	○	○	●	●	●	5

Table Note: ●adds a point on the score, ○adds no point on the score. The item “eligibility criteria” is not included in the final score.

The JBI (Checklists Critical assessment Tool) was employed to determine the quality of articles in randomized controlled trials, which revealed the absence of blinding of participants (19/19), treatment providers (19/19), and outcome evaluators (15/19). This may lead to performance or detection bias, especially for subjective outcome measures (Supplementary Table S1).

Using ROB two to assess methodological quality and bias in the included studies (Supplementary Figure S1A, B). The majority of studies were assessed as “some concerns”, with only three studies being at low risk of bias (Nelson et al., 1994; Liu-Ambrose et al., 2004; Bocalini et al., 2009) and two studies at high risk (Milliken et al., 2003; Maddalozzo et al., 2007). The main source of concern was potential bias due to the selection of the reported result. Two studies were at high risk of bias due to the randomization process (Milliken et al., 2003; Maddalozzo et al., 2007). These issues may be due, in part, to lack of clarity in reporting rather than study conduct, as many studies did not publish a protocolor analysis plan or there was a lack of clarity in reporting method of randomization. Other sources of concern were potential deviations from the stated interventions (Nichols KPN et al., 1995; Hartard et al., 1996; Rhodes et al., 2000; Maddalozzo et al., 2007) and Only one study (Chilibeck et al., 2002) had some concerns about the risks in the outcome measures. One study (Pruitt et al., 1995) has some concerns about missing outcome data of the included study, and one study (Milliken et al., 2003) shows a high risk.

### 3.4 Network meta-analysis

#### 3.4.1 LS BMD

**FIGURE 2:**
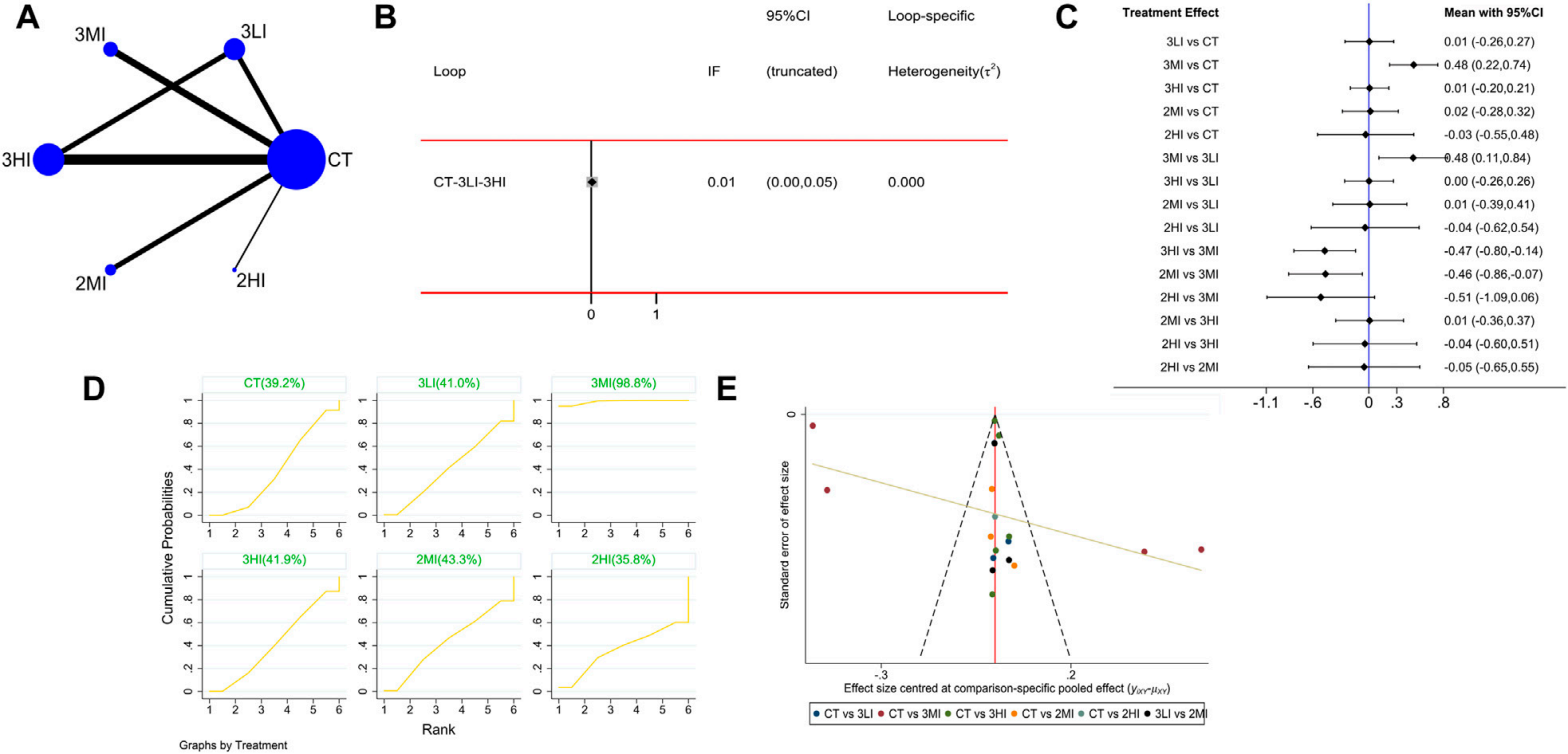
Network meta-analysis results forlumbar spine (LS) BMD. **(A)** Network evidence diagram; **(B)** loop inconsistency test; **(C)** forest plot; **(D)** the figure of cumulative probability ranking; **(E)** funnel plot.

A total of 16 studies with 722 subjects were included in this part. The network evidence diagram is shown as Figure 2A. The loop inconsistency test manifested that the 95%CI of the loop in consistency factor (IF) contained 0, indicating good consistency between the direct and indirect evidence in Figure 2B. The pairwise comparison of forest plot in Figure 2C showed that 3MI was superior in improving LS BMD compared to the control group, and the difference was statistically significant [WMD = 0.48, 95%CI(0.22,0.74),*p* < 0.05]; 3MI was superior to 2MI [WMD = −0.46,95%CI(−0.86, −0.07),*p* < 0.05], 3LI [WMD = 0.48,95%CI(0.11,0.84),*p* < 0.05], and 3HI [WMD = −0.47,95%CI(−0.80, −0.14),*p* < 0.05], which indicated that RT at moderate intensity was better than the other two intensities and that high frequency training at moderate intensity was better than low frequency training. Moreover, the SUCRA results in Figure 2D showed the highest SUCRA value appeared at 3MI (98.8%). Based on the above research, 3MI may be the optimal option.

#### 3.4.2 FN BMD

**FIGURE 3:**
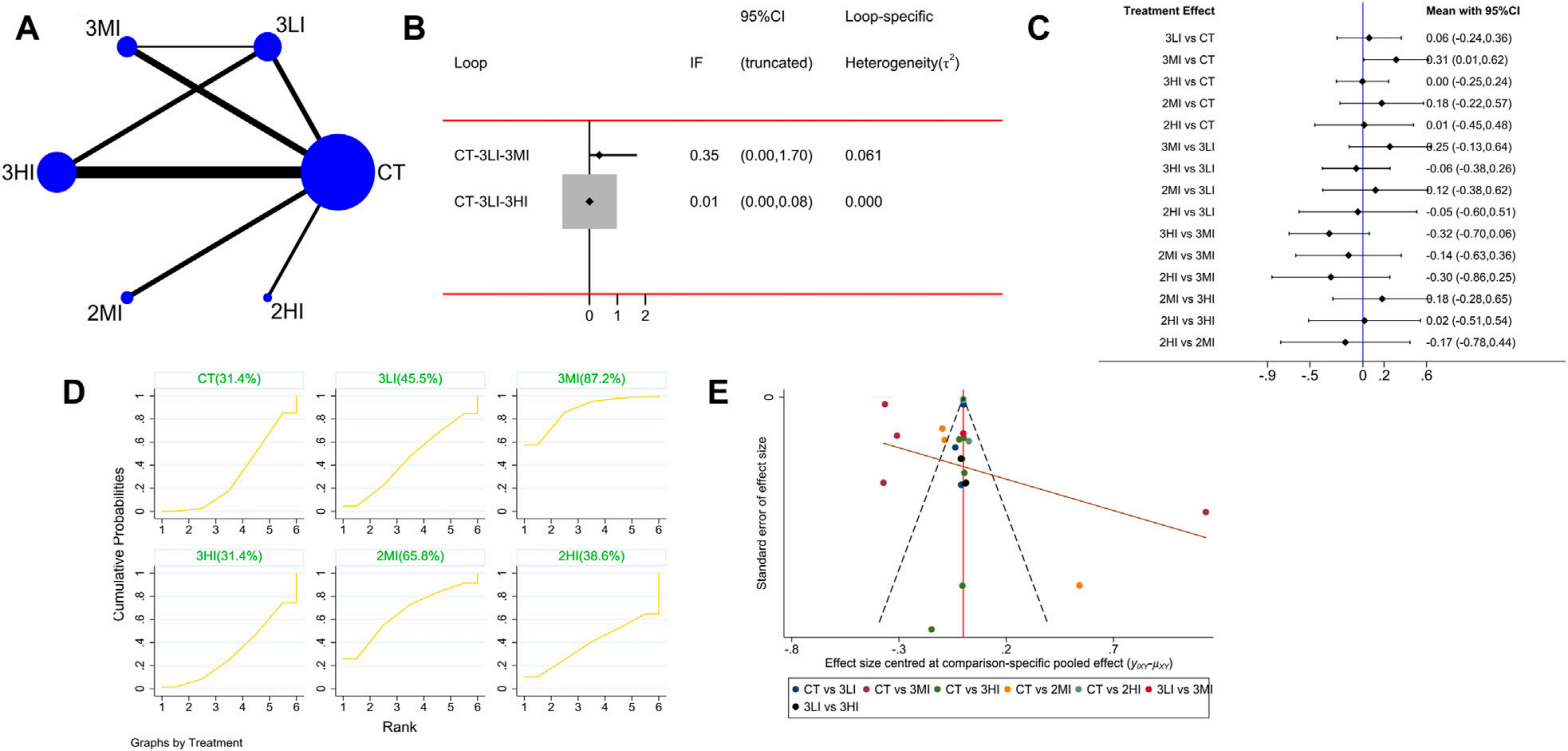
Network meta-analysis results for femoral neck (FN) BMD. **(A)** Network evidence diagram; **(B)** loop inconsistency test; **(C)** forest plot; **(D)** the figure of cumulative probability ranking; **(E)** funnel plot.

A total of 19 studies with 767 subjects were included. The network evidence diagram is shown as Figure 3A. The loop inconsistency test in Figure 3B indicated that 95% CI of the loop inconsistency factor (IF) contained 0, indicating good consistency between the direct and indirect evidence. The pairwise comparison of the forest plot in Figure 3C manifested that 3MI was effective in improving FN BMD compared to the control group, and the difference was statistically significant [WMD = 0.31,95% CI (0.01,0.62), *p* < 0.05]; in the pairwise comparison of the other groups, although 3MI had a significant advantage on FN BMD, the difference was not statistically significant (*p* > 0.05). Besides, the cumulative probability ranking in Figure 3D showed that 3MI ranked first (SUCRA value of 87.2%). For the above reason, 3MI may be the optimal option to improve FN BMD.

#### 3.4.3 TH BMD

**FIGURE 4:**
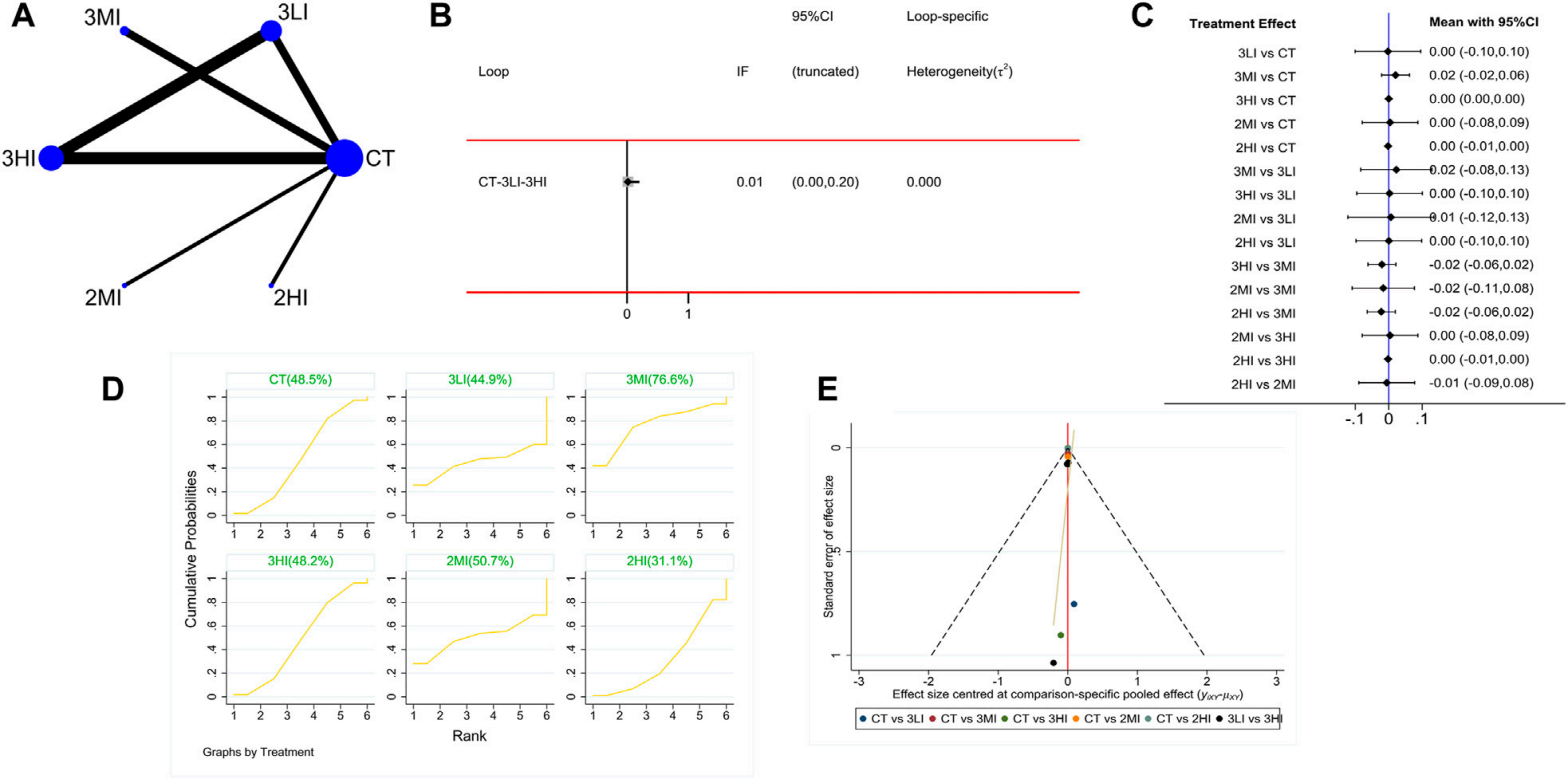
Network meta-analysis results for total hip (TH) BMD. **(A)** Network evidence diagram; **(B)** loop inconsistency test; **(C)** forest plot; **(D)** the figure of cumulative probability ranking; **(E)** funnel plot.

A total of eight studies with 282 subjects were included in this part. The network evidence diagram is shown as Figure 4A. The loop inconsistency test showed in Figure 4B that 95% CI of the loop inconsistency factor (IF) contained 0, indicating good consistency between the direct and indirect evidence. The forest plot in Figure 4C presented no statistical difference in the pairwise comparisons (*p* > 0.05). Meanwhile, the cumulative probability ranking in Figure 4D showed that 3MI ranked first (SUCRA value of 76.6%). Based on the above study, 3MI may be the best protocol to improve TH BMD.

#### 3.4.4 Troch BMD

**FIGURE 5:**
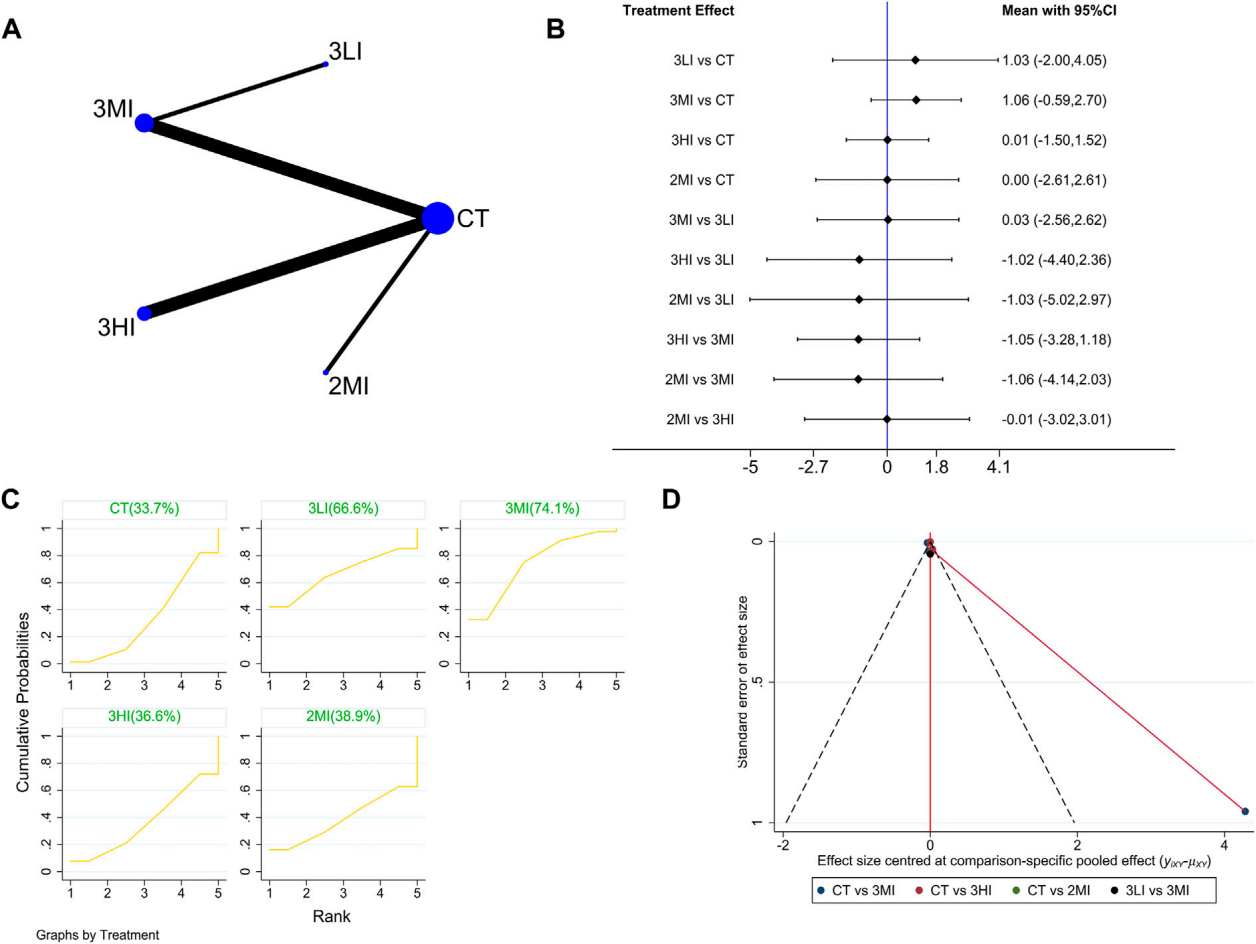
Network meta-analysis results forTroch BMD. **(A)** Network evidence diagram; **(B)** forest plot; **(C)** the figure of cumulative probability ranking; **(D)** funnel plot.

A total of eight studies with 393 subjects were included in this part. The network evidence diagram is shown in Figure 5A. The loop inconsistency test in Figure 5B showed good consistency between the direct and indirect evidence. The forest plot in Figure 5C indicated no statistically significant difference between the pairwise comparisons (*p* > 0.05). Meanwhile, the cumulative probability ranking in Figure 5D showed that 3MI ranked first (SUCRA value of 74.1%). To sum up, 3MI may be the best protocol to improve Troch BMD.

### 3.5 Publication bias

It can be seen from Figures 2E–5E that the funnel plot of each indicator was basically symmetrical and most of the points were in the upper part of the funnel, while only a few points of LS and FN BMD fell in the outer part of the funnel. The overall results revealed that publication bias was less likely contained in this study, but the interpretation of the results still needs to be treated with caution.

### 3.6 Sensitivity analysis

In this part, sensitivity analyses were also performed by removing studies with sample sizes less than 10 to verify the robustness of our results.

In regard to LS BMD, studies including Bemben-2000 and Maddalozzo-2000 were removed. The network evidence diagram and loop inconsistency test are shown in Supplementary Figure S2A, B. The pairwise comparisons in the forest plot (Supplementary Figure S2C) and the cumulative probability ranking (Supplementary Figure S2D) were not significantly different from the overall results. Hence, it can be concluded that our results are stable and reliable.

In regard to FN BMD, studies including Bemben-2000, Pruitt1995 and Maddalozzo-2000 were excluded. The network evidence diagram and loop inconsistency test are shown in Supplementary Figure S3A, B. The pairwise comparisons in the forest plot (Supplementary Figure S3C) and the cumulative probability ranking (Supplementary Figure S3D) were not significantly different from the overall results. Therefore, it can be concluded that the results are stable and reliable.

With respect to TH BMD, studies including Bemben-2000 and Maddalozzo-2000 were removed. The network evidence diagram and loop inconsistency test are shown in Supplementary Figure S4A, B. The pairwise comparisons in the forest plot (Supplementary Figure S4C) and the cumulative probability ranking (Supplementary Figure S4D) were not significantly different from the overall results. Consequently, it can be concluded that our results are reliable.

### 3.7 Subgroup analysis

Furthermore, considering that differences in intervention time might have an impact on the results of this study, subgroup analyses of LS BMD and FN BMD were performed on the basis of different intervention time. Two subgroups were set up: Intervention time ≤48 weeks group and intervention time>48 weeks group. Whereas, TH BMD, Troch BMD was not available for subgroup analysis due to the number of included studies.

In intervention time ≤48 weeks group of LS BMD, the network evidence diagram and loop inconsistency test are shown in Supplementary Figure S5A, B. The results of the forest plot (Supplementary Figure S5C) showed that 3MI was superior to CT in improving LS BMD (*p* < 0.05), and was also superior to 3LI and 3HI (*p* < 0.05). For RT at moderate intensity, 3MI was better than 2MI in improving BMD, with a statistically significant difference (*p* < 0.05). In intervention time>48 weeks group, the network evidence diagram and loop inconsistency test are shown in Supplementary Figure S6A, B. The results of the forest plot (Supplementary Figure S6C) showed that the difference was not statistically significant in either two comparisons, despite of the relative advantage of 3MI (*p* > 0.05). Additionally, the cumulative probability ranking results of both subgroups (Supplementary Figures S5D, 6D) showed that 3MI was the best option to improve LS BMD.

Network evidence diagram and loop inconsistency test for both subgroups in FN BMD are shown in Supplementary Figures 7A–B, 8A–B, respectively. Forest plot results (Supplementary Figure S7C) in intervention time ≤48 weeks group showed that resistance training at moderate intensity (either 3MI or 2MI) was superior to other RT protocols in improving FN BMD, with a statistically significant difference (*p* < 0.05). In contrast, in the subgroup with intervention time>48 weeks, the results of forest plot (Supplementary Figure S8C) showed no statistically significant difference between the groups in pairwise comparisons (*p* < 0.05). The results of the cumulative probability ranking (Supplementary Figures S7D, 8D) showed that the best protocol with intervention duration within 1 year (≤48 weeks) was 3MI, whereas the best protocol with intervention time>48 weeks was 2HI (68.0%).

## 4 Discussions

This study provides evidence for selecting optimal resistance training for postmenopausal women. As is known to all, the effect of RT on BMD is related to resistance form, resistance intensity, training frequency and training duration (Sañudo et al., 2017). This Meta-analysis explored the effect of resistance training at different intensities and frequencies on BMD at different sites (lumbar spine, femoral neck, total hip and trochanter) in postmenopausal women and performed a subgroup analyses based on intervention time to find the optimal combination at different intervention time through comparison. The network Meta-analysis showed that resistance training at moderate intensity for 3 days a week (3MI), was relatively effective in improving LS, FN, TH and Troch BMD in postmenopausal women, especially LS and FN BMD which was more significant.

With regard to training intensity, numerous studies have hypothesized that there was a threshold at which BMD values increased with the increase of training intensity, and beyond which it didn’t (Vainionpää et al., 2006). However, some studies proposed that excessive training intensity can even lead to a decrease in BMD and negatively affect bone health. This may be caused by the fact that prolonged high intensity training disrupts the endocrine system, which interferes with the hypothalamic-pituitary-gonadal axis and indirectly inhibits the production and release of estrogen from the ovaries, further reducing the concentration of estrogen in the blood and weakening bone formation.

Results of network Meta-analysis showed that moderate intensity was more superior in increasing BMD compared to high intensity, and the cumulative probability ranking also showed that moderate intensity was the optimal solution, similar to the results of studies such as MICHL (Verschueren et al., 2004). The possible reason was that the strain of bone generated by the stimulation of mechanical loading at moderate intensity led to increasing activity of osteoblasts, greater bone formation than bone resorption, and continuous accumulation of bone minerals, which all resulted in the increased BMD. Hence, it can be extrapolated that moderate intensity training may be just enough to produce strain on the bone without excessive stress causing subtle damage, and thus it stimulated osteoclast proliferation and increased bone formation. Furthermore, some studies revealed that low intensity training had an insufficiently significant effect on BMD increase (Kistler-Fischbacher et al., 2021). The reason for this may be that lower loads mostly did not reach the stress threshold of bone strain and not effectively stimulate bone tissue.

In this paper, the training frequency was also considered, and research results revealed that 3 days/week of moderate intensity training (3MI) was mostly better than 2 days/week (2MI), and this difference was more pronounced in LS BMD. This was because the frequency of loading was the main influence factor that induced a bone adaptive response that could directly affect bone formation. PINHEIRO (Borba-Pinheiro et al., 2016) demonstrated that resistance training performed 3 times/week by postmenopausal women was better than 2 times/week in improving LS and FN BMD, which was generally consistent with the results of this study. In terms of this study, sensitivity analysis was conducted by excluding studies with a single group sample size of less than 10, and the results similarly presented that 3MI was the optimal choice. It suggested that our findings are more reliable.

Previous studies have shown that exercise produces adaptive bone development only when it achieved cumulative time and volume. The bone reconstruction cycle generally takes 3–4 months, and the bone needs 7–9 months to achieve a new stable level of bone volume after alteration (Fuchs et al., 2001); hence, clinical trials of ≥6 months were selected for this study on the included literature in order to make the results stable and realistic. Additionally, considering the influence of intervention time on the effect of RT, subgroup analyses were performed. Two subgroups were set up: Intervention time ≤48 weeks group and intervention time>48 weeks group. Subgroup analyses results showed that for LS and FN BMD, 3MI protocol with intervention duration within 1 year (≤48 weeks) had a significant advantage over other interventions, while this advantage was no longer significant with the intervention duration of more than 1 year (>48 weeks). The reason may be related to the reduced sensitivity of bone to stress stimuli due to prolonged adoption of exercise at the same intensity, which led to slower bone plasticity building (Benedetti et al., 2018). Apart from this, prolonged endurance training interfered with the secretion of hormones and other functions of the hypothalamus, resulting in low levels of sex hormones or a severe lack of them, breaking the balance of osteoblast and osteoclast activity and causing bone resorption greater than bone formation. In conclusion, when designing resistance training prescriptions for postmenopausal women, the duration and intensity of exercise should be fully considered. As a consequence, when developing training program, the training duration should not be too long unless a new strain distribution forms, and either progressive resistance or alternating exercises at different intensities can be selected.

The results of this meta analysis are helpful to refine the exercise prescription of resistance exercise and provide evidence for improving bone mineral density in postmenopausal women. There are a large number of postmenopausal women in the world and a lot of money is spent on the fight against osteoporosis. The development of resistance movement does not need to invest expensive equipment and large areas, which can save a lot of medical expenses. In addition, resistance exercise has good safety, and participants will feel the pleasure of dopamine secretion during exercise (Nybo et al., 2003). To sum up, resistance exercise is of great clinical significance.

## 5 Limitations

Although we included all interventions in this network meta-analysis to obtain comprehensive results, the study had certain limitations. Firstly, the population included in the study was not identical in terms of daily exercise habits and exercise sites for RT, and most RCTs did not describe details of the randomized scheme, allocation concealment and blinding of subjects and researchers with regard to article quality assessment, which could lead to potential heterogeneity. Secondly, this analysis included only studies that quantified exercise intensify using 1RM. However, only 19 studies were qualified through screening under the precise classification. The lack of direct evidence may limit the reliability of the results and it’s necessary to treat it with caution. Finally, since the included studies ended up being available only in English, there may be language bias.

## 6 Conclusion

In conclusion, moderate intensity and high frequency (at 65%–80% 1RM, 3 days/week) may be the optimal training protocol to improve LS, FN, TH and Troch BMD in postmenopausal women. Considering the effect of the exercise cycle, it is recommended that the same training protocol should preferably last no longer than 1 year in order to reasonably regulate bone adaptation and produce new strain distribution. However, due to study limitations, the conclusion drawn in this paper need to be further validated by more high-quality studies.

## Data Availability

The original contributions presented in the study are included in the article/Supplementary Material, further inquiries can be directed to the corresponding author.
