# Supplementary material for: Comparative efficacy different resistance training protocols on bone mineral density in postmenopausal women: A systematic review and network meta-analysis
Source: Front Physiol. 2023 Feb 7;14:1105303. doi: 10.3389/fphys.2023.1105303 (PMC9941565; doi:10.3389/fphys.2023.1105303)

**Supplementary Material**

Zhenyu Wang^1†^,Xingchun Zan^2†^,Yongjie Li^3†*^,Yue Lu^4^,Yuan Xia^1^,Xinyong Pan^1^

^1^School of Health Sciences ,Wuhan Sports University,Wuhan, China;

^2^ Department of Rehabilitation Medicine,Hefei Second People's Hospital,Hefei,China.

^3^Department of Rehabilitation Medicine, Beijing Jishuitan HospitalGuizhouHospital

,Guiyang,China

^4^Department of Rehabilitation Medicine, Hubei Provincicial Hospital of Integrated Chinese and Western Medicine ,Wuhan, China;

* Correspondence:
Corresponding Author:Yongjie Li,yjl20210201@163.com

†These authors have contributed equally to this work

**Table of Contents**

**Table 1.**JBI critical appraisal checklist for randomized controlled trials

**Figure1.**Version 2 of the Cochrane tool for assessing risk of bias in randomised trial**.**A:Risk of Bias summary graph. B:Risk of bias graph.

**Figure2.** Sensitivity analysesof LS BMD.A: Network evidence diagram.B: loop inconsistency test.C:Forest plot of sensitivity analysis.D: The figure of Cumulative probability ranking.

**Figure3.** Sensitivity analysesof FN BMD.A:Network evidence diagram.B:loop inconsistency test.C:Forest plot of sensitivity analysis.D: The figure of Cumulative probability ranking.

**Figure4.** Sensitivity analysesof TH BMD.A:Network evidence diagram.B:loop inconsistency test.C:Forest plot of sensitivity analysis.D: The figure of Cumulative probability ranking.

**Figure5.** Subgroup analyses ofintervention time≤48weeks group of LS BMD. A:Network evidence diagram.B:loop inconsistency test.C:Forest plot of subgroup analysis.D: The figure of Cumulative probability ranking.

**Figure6.** Subgroup analyses ofintervention time＞48weeks group of LS BMD. A:Network evidence diagram.B:loop inconsistency test.C:Forest plot of subgroup analysis.D: The figure of Cumulative probability ranking.

**Figure7.** Subgroup analyses of intervention time≤48weeks group of FN BMD. A:Network evidence diagram.B:loop inconsistency test.C:Forest plot of subgroup analysis.D: The figure of Cumulative probability ranking.

**Figure8.** Subgroup analyses of intervention time＞48weeks group of FN BMD. A:Network evidence diagram.B:loop inconsistency test.C:Forest plot of subgroup analysis.D: The figure of Cumulative probability ranking.

**Table 1.**JBI critical appraisal checklist for randomized controlled trials

| Study | Q1 | Q2 | Q3 | Q4 | Q5 | Q6 | Q7 | Q8 | Q9 | Q10 | Q11 | Q12 | Q13 |
| --- | --- | --- | --- | --- | --- | --- | --- | --- | --- | --- | --- | --- | --- |
| Pruitt  1992(20) | U | N | Y | N | N | N | Y | Y | Y | Y | Y | Y | Y |
| Nelson1  1994(21) | Y | U | Y | N | N | Y | Y | Y | Y | Y | U | Y | Y |
| Nichols  1995(22) | Y | U | Y | N | N | Y | Y | N | Y | Y | U | Y | Y |
| Pruitt  1995(23) | Y | U | Y | N | N | Y | Y | U | Y | Y | Y | Y | Y |
| Hartard  1996(24) | U | N | Y | N | N | N | Y | Y | Y | Y | Y | Y | Y |
| Kerr  1996(25) | U | U | Y | N | N | N | Y | U | Y | Y | Y | Y | Y |
| Bemben  2000(26) | Y | U | Y | N | N | N | Y | U | Y | Y | Y | Y | Y |
| Maddalozzo  2000(27) | Y | U | Y | N | N | N | Y | Y | Y | Y | Y | Y | Y |
| Rhodes  2000(28) | Y | U | Y | N | N | N | Y | U | Y | Y | Y | Y | Y |
| Chilibeck  2002(29) | U | U | Y | N | N | N | Y | U | Y | Y | Y | Y | Y |
| Milliken  2003(30) | U | U | Y | N | N | N | Y | U | Y | Y | Y | Y | Y |
| Liu  2004(31) | Y | U | Y | N | N | Y | N | Y | Y | Y | Y | Y | Y |
| Siegrist  2006(32) | Y | U | Y | N | N | N | Y | Y | Y | U | Y | Y | Y |
| Maddalozzo  2007(33) | U | U | Y | N | N | N | Y | U | Y | Y | Y | Y | Y |
| Bocalini  2009(34) | Y | U | Y | N | N | N | Y | N | U | Y | Y | Y | Y |
| Chuin  2009(35) | Y | U | Y | N | N | N | Y | N | Y | Y | Y | Y | Y |
| Bemben  2010(36) | U | N | Y | N | N | N | Y | U | Y | Y | Y | Y | Y |
| Bocalini  2010(37) | Y | U | Y | N | N | Y | Y | U | Y | Y | Y | Y | Y |
| Marques  2012(38) | Y | U | Y | N | N | N | Y | Y | Y | Y | Y | Y | Y |
| Total Y | 14  (74%) | 0 | 19  (100%) | 0 | 0 | 4  (21%) | 18  (95%) | 7  (37%) | 18  (95%) | 18  (95%) | 17  (89%) | 19  (100%) | 19  (100%) |
| Total N | 0 | 3  (16%) | 0 | 19  (100%) | 19  (100%) | 15  (79%) | 1  (5%) | 3  (16%) | 0 | 0 | 0 | 0 | 0 |
| Total U | 5  (26%) | 16  （84%） | 0 | 0 | 0 | 0 | 0 | 9  (16%) | 1  (5%) | 1  (5%) | 2  (11%) | 0 | 0 |

Y=Yes, U=Unclear, N=No; JBI critical appraisal checklist for randomized controlled trials: Q1=Was true randomization used forassignment of participants to treatment groups? Q2=Was allocation to treatment groups concealed? Q3=Were treatment groups similar at baseline? Q4=Wereparticipants blind to treatment assignment? Q5=Were those delivering treatment blind to treatment assignment? Q6=Were outcome assessors blind to treatmentassignment? Q7=Were treatment groups treated identically other than the intervention of interest? Q8=Was follow-up complete, and if not, were strategies toaddress incomplete follow-up utilized? Q9=Were participants analyzed in the groups to which they were randomized? Q10=Were outcomes measured in thesame way for treatment groups? Q11=Were outcomes measured in a reliable way? Q12=Was appropriate statistical analysis used? Q13=Was the trial designappropriate, and any deviations from the standard RCT design (individual randomization, parallel groups) accounted for in the conduct and analysis of the trial?


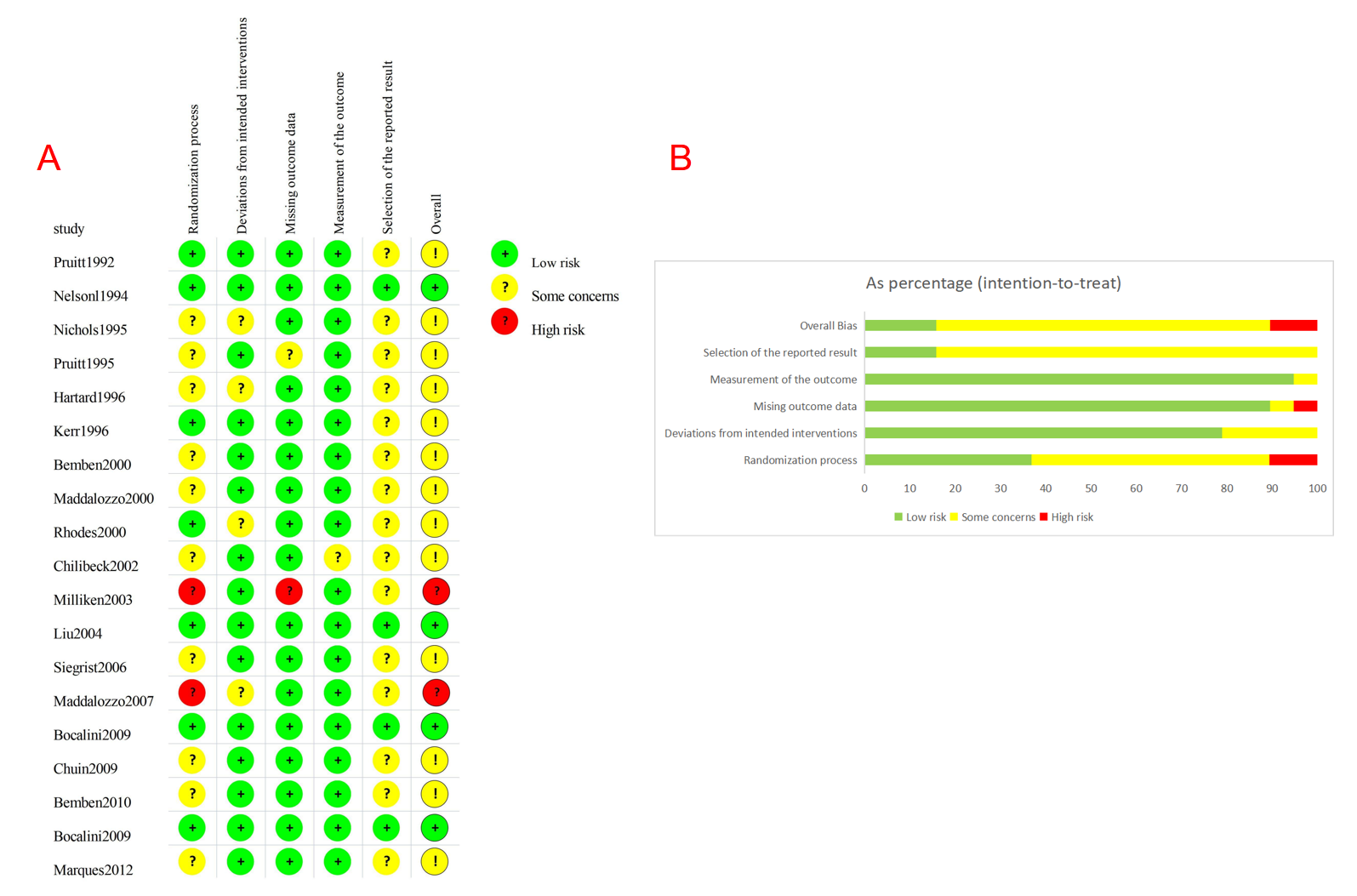


**Figure1.** Version 2 of the Cochrane tool for assessing risk of bias in randomised trial**.**A:Risk of Bias summary graph. B:Risk of bias graph.


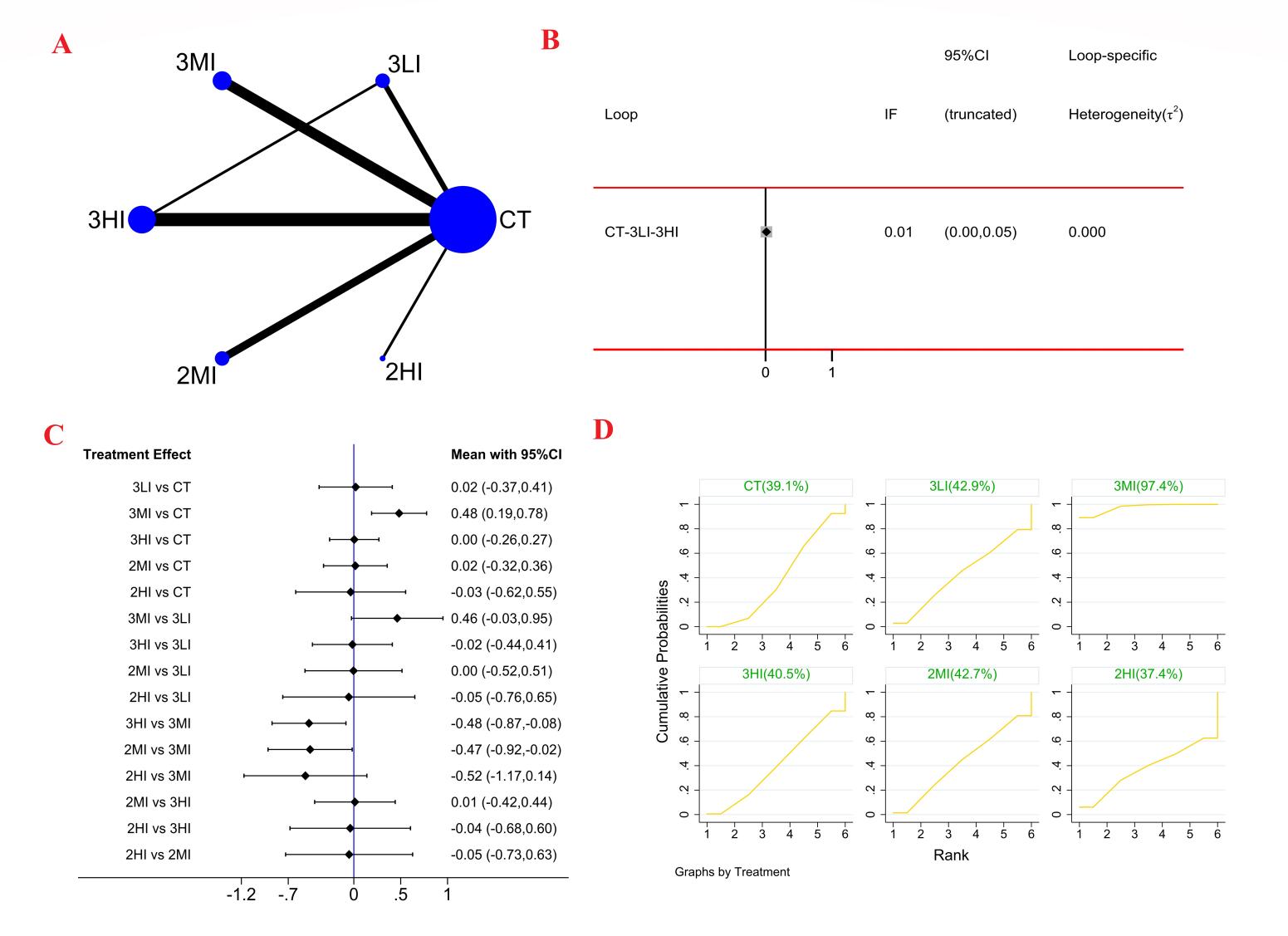


**Figure2.** Sensitivity analyses of LS BMD.A:Network evidence diagram.B:loop inconsistency test.C:Forest plot of sensitivity analysis.D: The figure of Cumulative probability ranking.


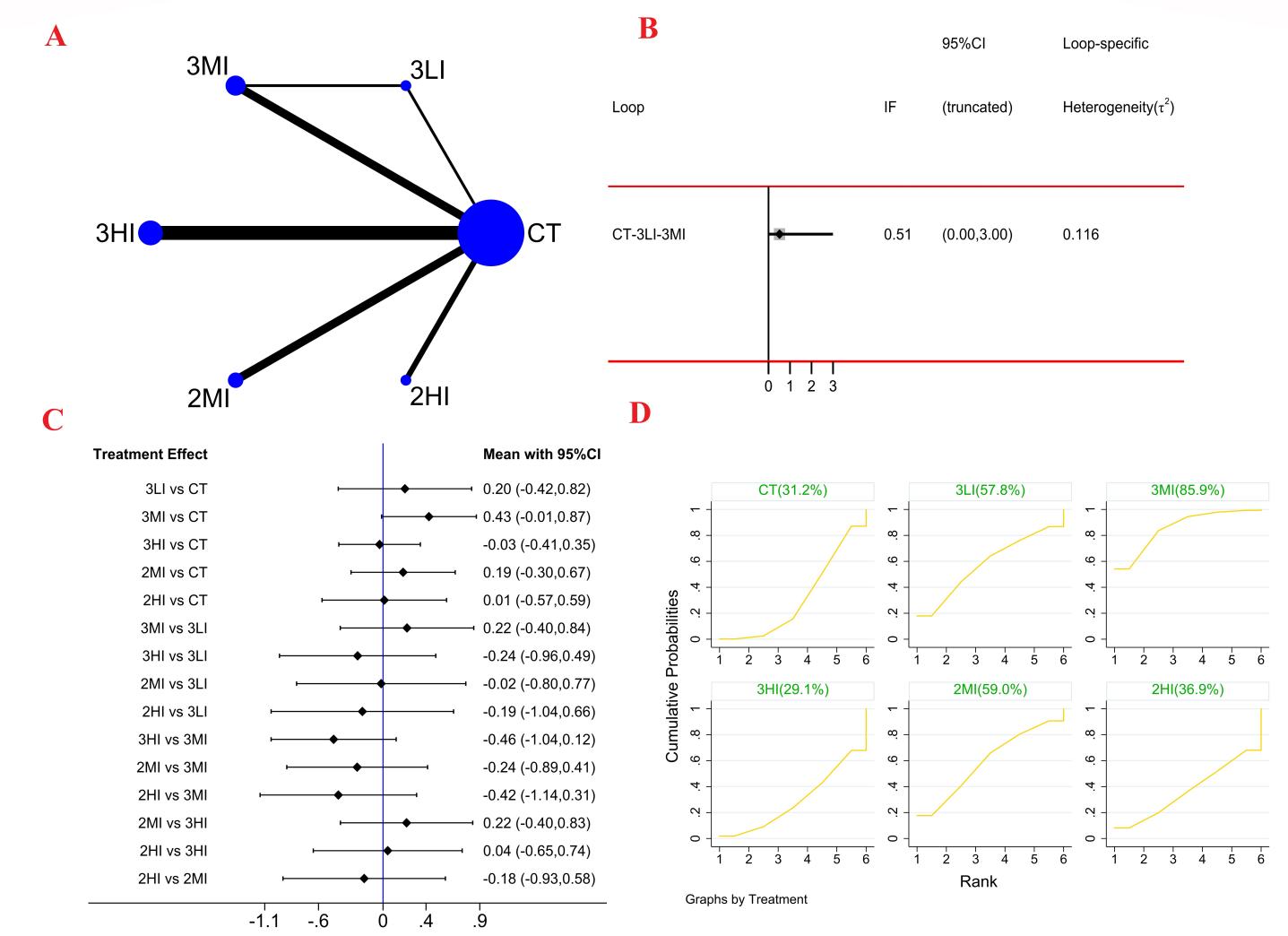


**Figure3.** Sensitivity analysesof FN BMD.A:Network evidence diagram.B:loop inconsistency test.C:Forest plot of sensitivity analysis.D: The figure of Cumulative probability ranking.


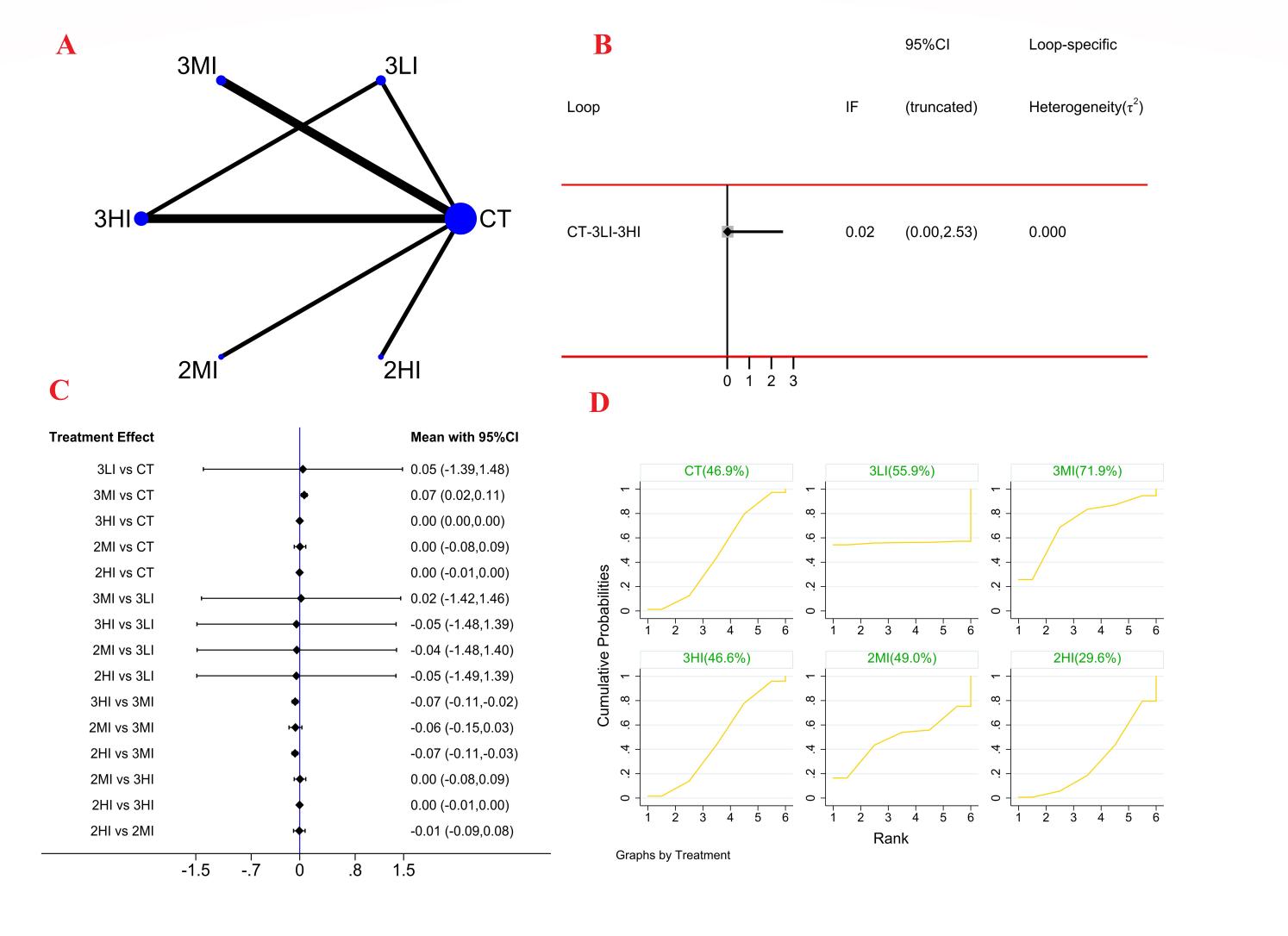


**Figure4.** Sensitivity analysesof TH BMD.A:Network evidence diagram.B:loop inconsistency test.C:Forest plot of sensitivity analysis.D: The figure of Cumulative probability ranking.


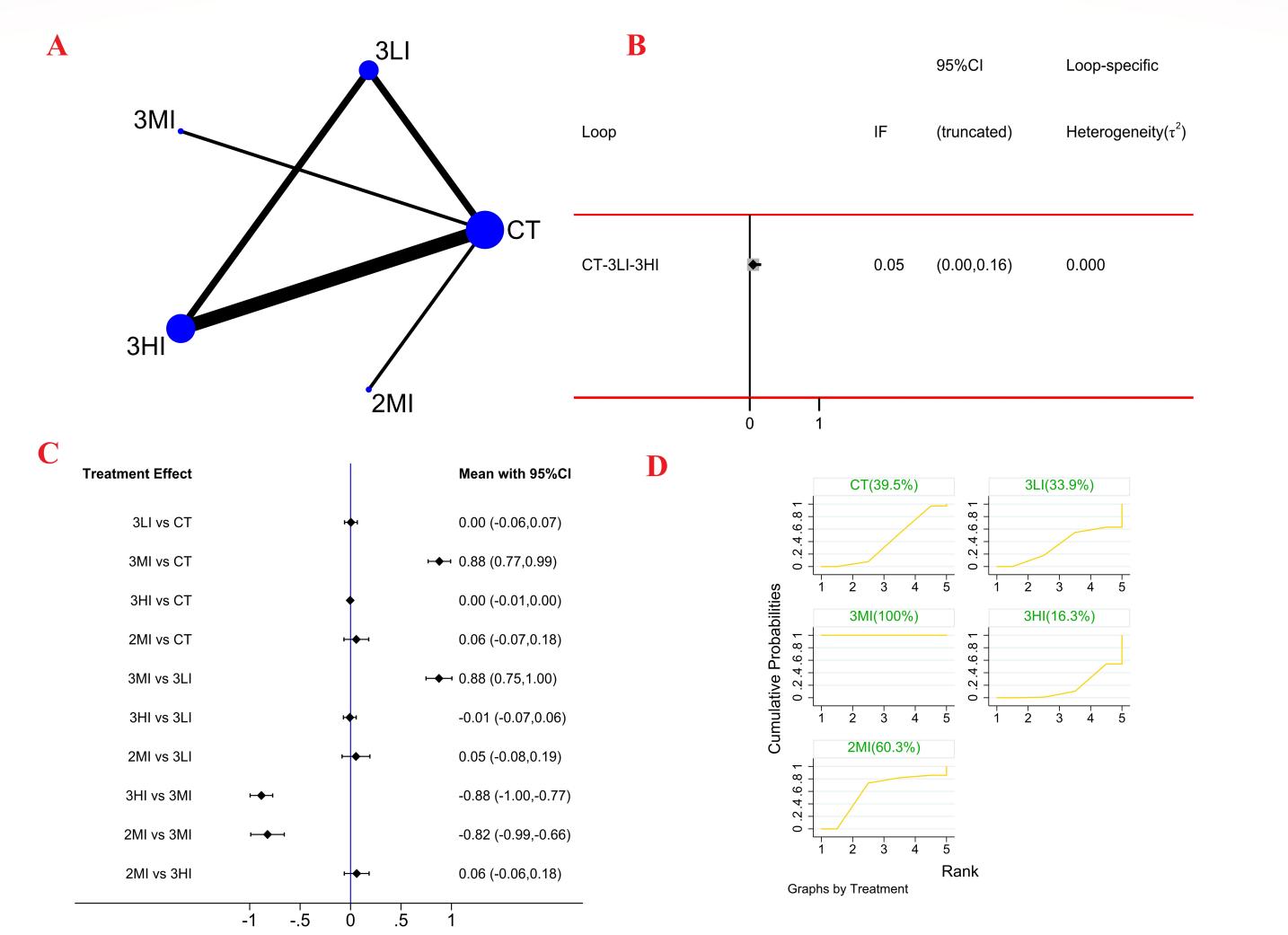


**Figure5.**Subgroup analyses ofintervention time≤48weeks group of LS BMD. A:Network evidence diagram.B:loop inconsistency test.C:Forest plot of subgroup analysis.D: The figure of Cumulative probability ranking.


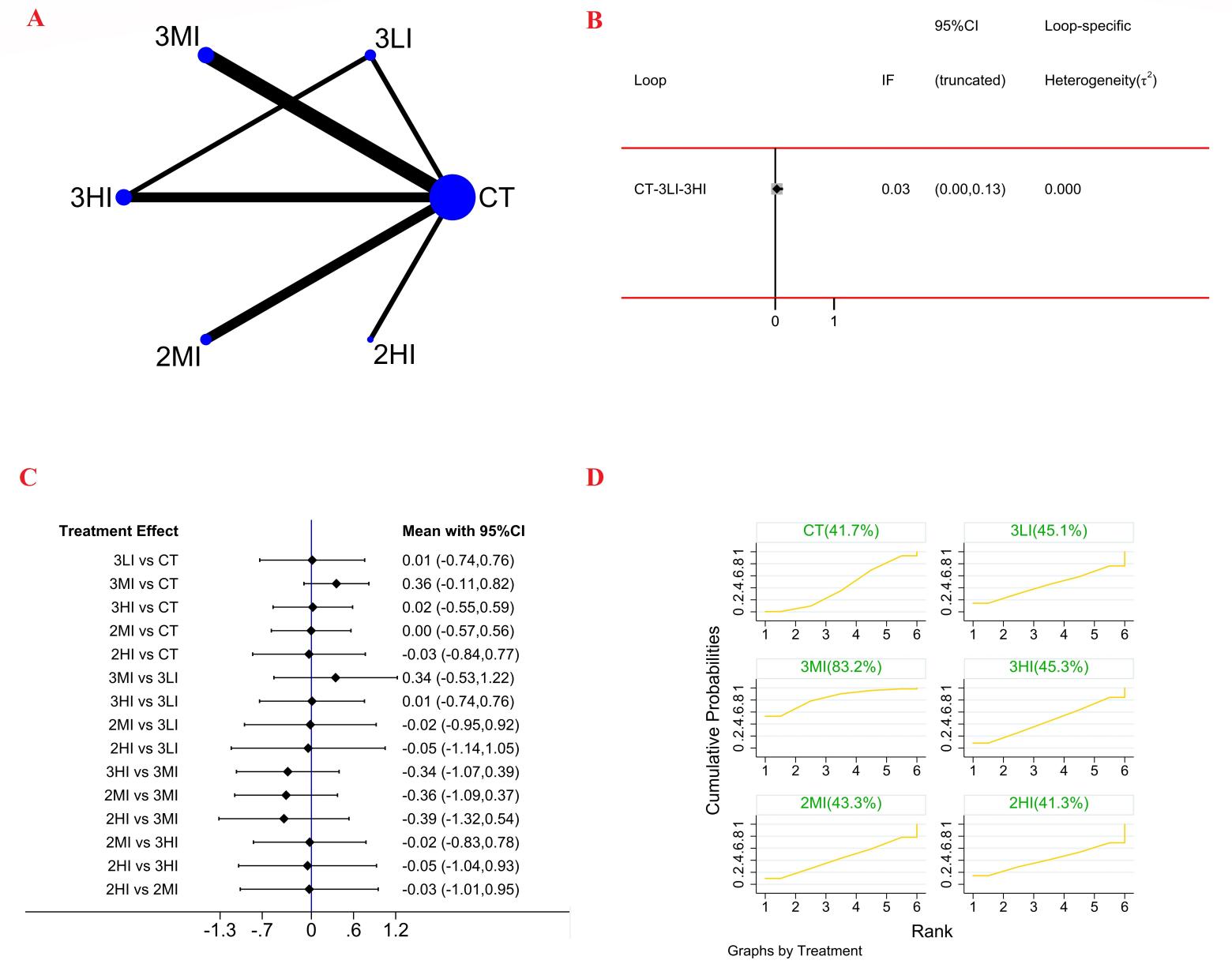


**Figure6.**Subgroup analyses ofintervention time＞48weeks group of LS BMD. A:Network evidence diagram.B:loop inconsistency test.C:Forest plot of subgroup analysis.D: The figure of Cumulative probability ranking.


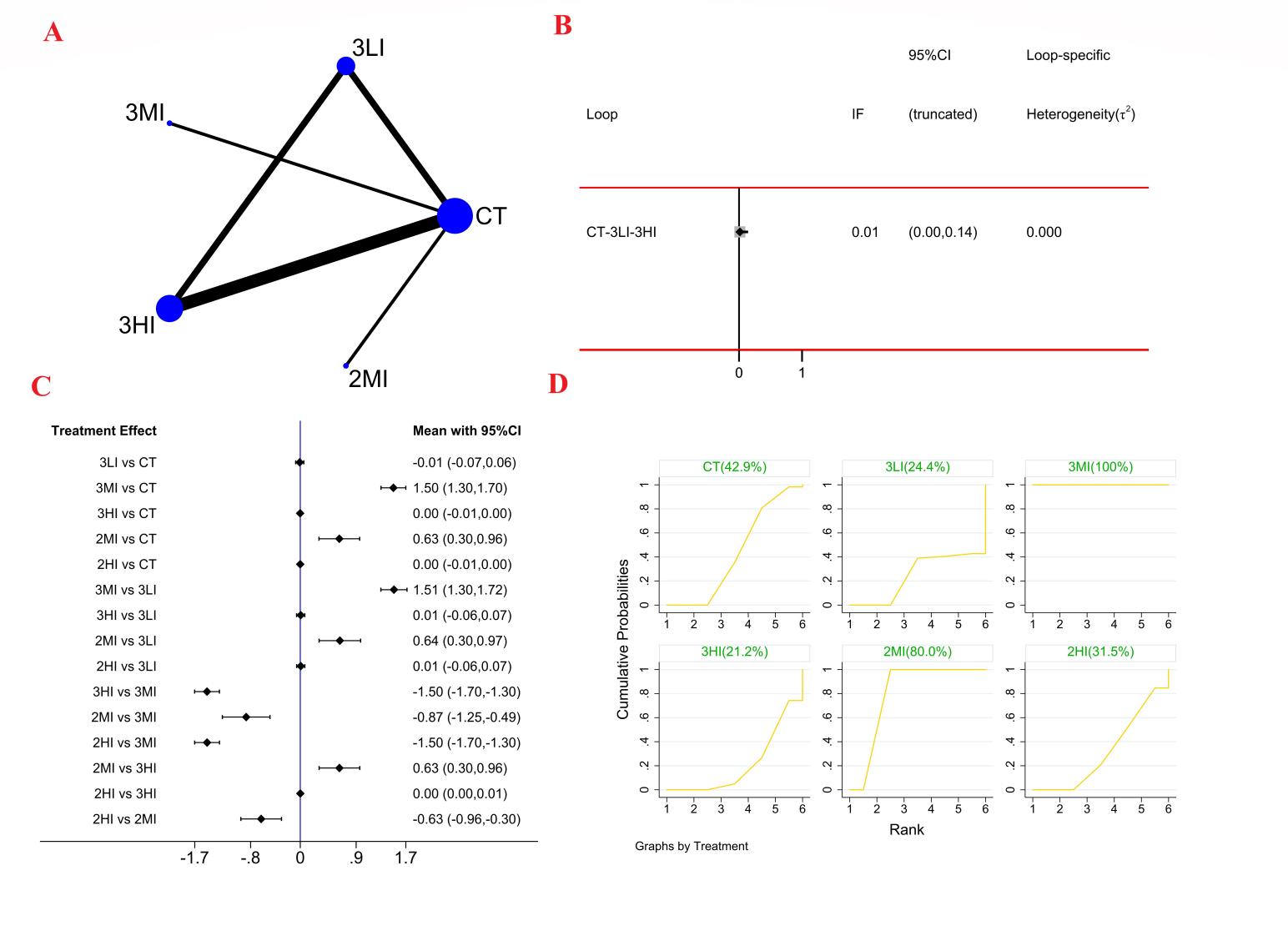


**Figure7.**Subgroup analyses of intervention time≤48weeks group of FN BMD. A:Network evidence diagram.B:loop inconsistency test.C:Forest plot of subgroup analysis.D: The figure of Cumulative probability ranking.


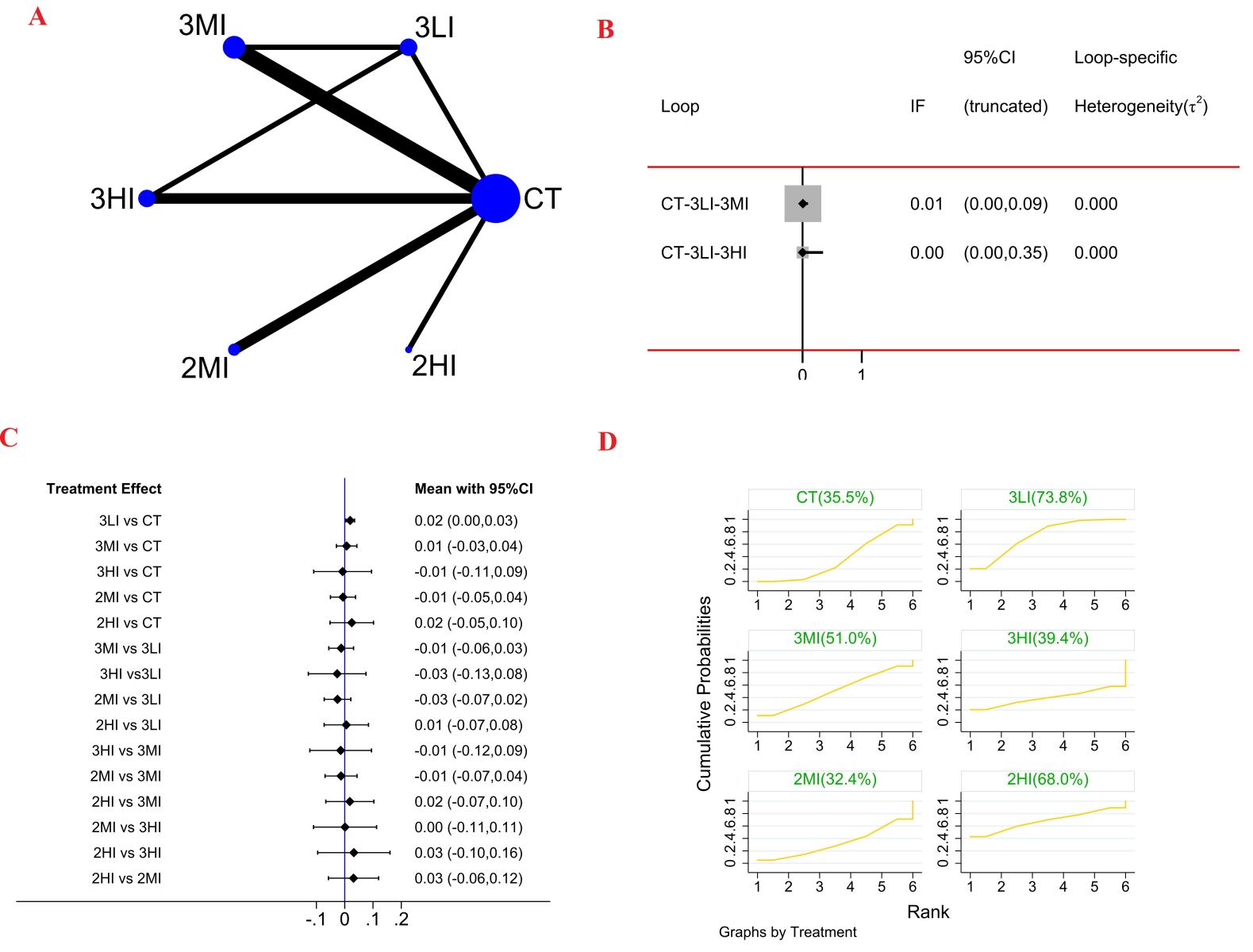


**Figure8.**Subgroup analyses of intervention time＞48weeks group of FN BMD. A:Network evidence diagram.B:loop inconsistency test.C:Forest plot of subgroup analysis.D: The figure of Cumulative probability ranking.


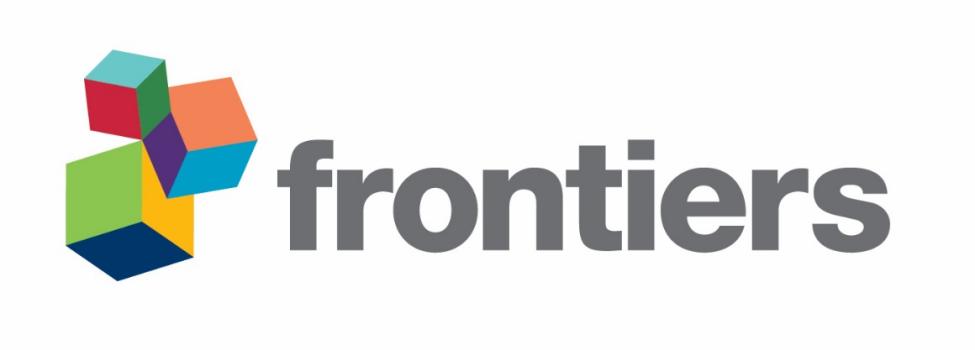

Supplement: Supplementary file 1 [file Table1.DOCX]
